# Circulating tumor DNA molecular analyses and real-world evidence outcomes of *FGFR2* amplified gastroesophageal cancers

**DOI:** 10.1093/oncolo/oyae061

**Published:** 2024-06-21

**Authors:** Bushra Shariff, Reagan M Barnett, Farshid Dayyani, Steven B Maron, Rory Mcgriskin, Samuel Klempner, Elifnur Yay Donderici, Nicole Zhang, Jude Masannat, Leylah M Drusbosky, Rutika Mehta

**Affiliations:** H. Lee Moffitt Cancer Center, Tampa, FL, USA; Guardant Health, Redwood City, CA, USA; University of California Irvine, Irvine, CA, USA; Memorial Sloan Kettering Cancer Center, New York, NY, USA; Memorial Sloan Kettering Cancer Center, New York, NY, USA; Massachusetts General Hospital, Boston, MA, USA; Guardant Health, Redwood City, CA, USA; Guardant Health, Redwood City, CA, USA; Guardant Health, Redwood City, CA, USA; Guardant Health, Redwood City, CA, USA; H. Lee Moffitt Cancer Center, Tampa, FL, USA

**Keywords:** *FGFR2* amplification, gastroesophageal cancer, liquid biopsy, real-world data

## Abstract

**Purpose:**

In addition to the existing biomarkers HER2 and PD-L1, *FGFR2b* has become an area of interest for the development of new targeted-based treatment. Given that clinical evaluation of *FGFR2* targeted therapy is underway, we sought to elucidate the genomic landscape of *FGFR2*_amp_ in gastroesophageal cancer (GEC) using a circulating tumor DNA (ctDNA) platform.

**Materials and Methods:**

We retrospectively evaluated the Guardant Health database from 2017 to 2022 for patients with GECs with Guardant360 ctDNA next-generation sequencing (NGS) performed. We assessed co-occurring genetic alterations for patients who harbored *FGFR2*_amp_ versus *FGFR2*_null_. We also explored real-world evidence database with Guardant Health, publicly available genomic databases (MSK cohort using cBioPortal), and pooled clinical data from large-volume cancer centers for *FGFR2*_amp_ GECs.

**Results:**

Less than 4% of patients with GEC in the Guardant Health database were identified to be *FGFR2*_amp_. The most commonly co-occurring gene mutations were *TP53*, *CTNNB1*, *CDH1*, and *RHOA.* Upon interrogation of the MSK cohort, these same genes were not significant on tissue NGS in the *FGFR2*_amp_ cohort of GEC. In the pooled institutional cohort, we noted that *FGFR2*_amp_ tumors were most commonly involving the gastroesophageal junction (GEJ). The overall survival of these patients was noted at 13.1 months.

**Conclusion:**

*FGFR2* is a validated target in GECs, and the contexture of *FGFR2*_amp_ will be important in defining patient subgroups with responses to FGFR2-directed therapy. Using ctDNA to provide a more detailed genomic landscape in patients with GECs will allow the advancement of targeted therapy in the near future for these aggressive cancers.

## Teaser

FGFR2 overexpression or amplification has become an area of interest for the development of new targeted-based treatment. This article focuses on the genomic landscape of *FGFR2* amplified gastroesophageal cancers.

**Implications for Practice**

*FGFR2* amplification is a marker of poor prognosis in gastroesophageal cancers (GECs), and clinical trials are underway to study FGFR2 inhibitors in GECs. The use of a circulating tumor DNA (ctDNA) platform has offered the advantage to study the genomic alterations at different time points during the treatment of cancer without necessarily needing repeat biopsies. This study highlights how the ctDNA platform can be used to identify GECs with *FGFR2* amplification and describes the genomic alterations in *FGFR2*_amp_ and *FGFR2*_null_ GECs. The spectrum of co-occurring mutations was different when *FGFR2*_amp_ cases were interrogated from tissue testing. There was a predominance of cell cycle pathway genes in the co-occurring mutations, which can potentially be harnessed for future combination treatment strategies. This is even more important as FGFR2 inhibitors make their way in the GEC treatment landscape.

## Introduction

The fibroblast growth factor (FGF) pathway, including its receptors (FGFRs), regulates a broad spectrum of biological functions related to carcinogenesis. FGFRs 1-4 are transmembrane tyrosine kinase receptors with an extracellular domain for FGF ligand binding and an intracellular region that contains a tyrosine kinase motif and carboxy-terminal tail. The dimerization of the complex of FGF, FGFR, and heparin sulfate proteoglycans induces transphosphorylation of intracellular region of FGFRs. This leads to downstream signaling primarily through MAPK/PI3K/Akt pathway or others such as STAT-dependent signaling. Mutations, amplifications, and/or translocations in this pathway have been directly linked to oncogenesis.^1,2^ Different *FGFR* alterations are seen in various cancer types such as gastric (GC), ovarian, bladder, endometrial, and lung cancers.^3^

Amplification or overexpression of the *FGFR2* gene has been shown to enhance constitutive activation of the receptor and has been reported in approximately 4% of gastroesophageal cancers (GECs).^4,5^*FGFR2* undergoes alternative splicing in the third immunoglobulin domain, leading to 2 different isoforms of the FGFR2 receptor—FGFR2b and FGFR2c, with different FGF ligand binding. Amplification and overexpression of the *FGFR2b* splice variant have been linked to the deletion of a proximal coding exon causing persistent activation of the FGFR2 receptor, promoting oncogenesis.^1,6-9^ Studies evaluating the impact of *FGFR2* amplification in gastric adenocarcinoma showed an association with lymph node metastases (Odd’s ratio [OR] 3.93, *P* < .00001), poor differentiation (OR 2.36, *P* < .04) and worse prognosis/survival (HR 2.09, *P* < .00001); these did not, however, demonstrate an increased rate of tumor invasion.^9,10^

Given aberrant *FGFR2* signaling and its role in oncogenesis, FGFR2 inhibitors have become an attractive new therapeutic target. FGFR inhibitors have been approved for locally advanced and metastatic cholangiocarcinoma and urothelial cancers. In a randomized Phase II study, patients with advanced GC and gastroesophageal junction (GEJ) adenocarcinoma with immunohistochemical expression of FGFR2b or *FGFR2* amplification via circulating tumor DNA (ctDNA) were treated with chemotherapy +/− bemarituzumab, a recombinant monoclonal antibody against FGFR2b.^11^ The overall survival (OS) favored bemarituzumab (19.2 months vs 13.5 months for placebo [HR 0.60, 95% CI 0.38-0.94]) in the post hoc analysis with an additional long-term follow-up of 12.5 months.

The utility of ctDNA has significantly evolved in cancer therapeutics and is now included in various guidelines for the identification of molecular targets in a relatively noninvasive way, monitoring of disease and response to treatment as well as assessing residual disease especially in locally advanced stage settings.^12^ ctDNA has been shown to be affected by systemic and local treatments—chemotherapy, radiation, and immunotherapy. Given the ongoing evolution of FGFR2-directed approaches, we sought to characterize the genomic landscape of *FGFR2*_amp_ GECs in an effort to elucidate possible other co-occurring targetable mutations that would augment therapy selection for metastatic disease. In this study, we interrogated multiple cohorts of patients with GECs for *FGFR2* amplifications and sought to describe the genomic landscape of these tumors using ctDNA or tissue next-generation sequencing (NGS) and survival characteristics in comparison to a cohort of patients without *FGFR2* amplification.

## Materials and methods

This study was performed after obtaining appropriate institutional review board approvals from each participating institution. De-identified research datasets generated by Guardant Health are approved by the Advarra IRB with a waiver of consent. The Guardant INFORM database is a fully de-identified database that complies with sections 164.514(a)-(n)1ii of the US Health Insurance Portability and Accountability Act regarding the determination and documentation of statistically de-identified data. Retrospective analysis of the Guardant Health database is IRB-approved by Advarra Pro00034566.

**Figure 1.**
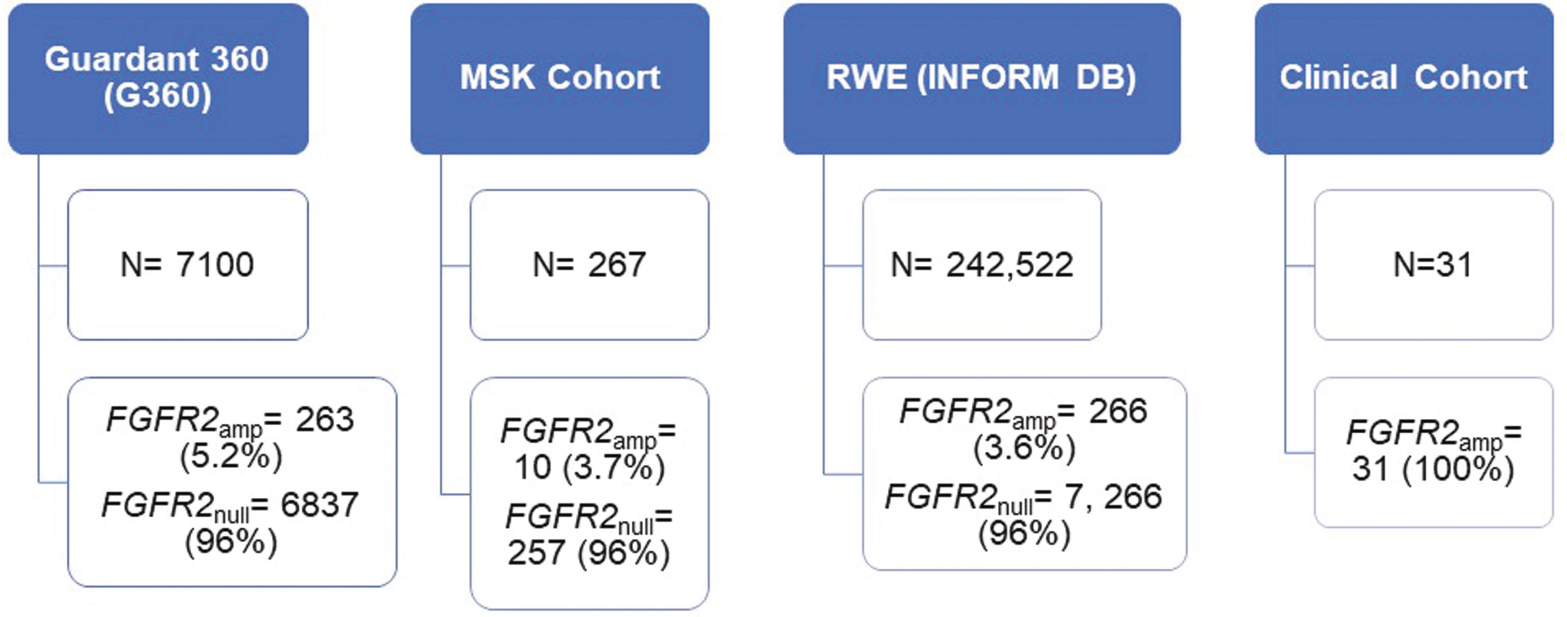
Outline of 4 different cohorts analyzed—Guardant 360 database, MSK cohort (cBioPortal), Guardant INFORM DB, and multi-institutional clinical cohort and number of *FGFR2*_amp_ GECs per cohort.

Four different data sets were analyzed for the purpose of this study as outlined in Figure 1.

### Guardant health database

We queried the Guardant Health genomic database from 2017 to 2022 for patients with advanced esophageal (EAC), GC, or GEJ adenocarcinomas, as reported on the test requisition form (TRF), who had ctDNA NGS (Guardant360 [G360], Redwood City, CA) performed as part of routine clinical care. Information regarding the patient’s timing of G360 testing was collected from the TRF as “newly diagnosed” or “not responding to therapy.”

Variants of unknown significance, synonymous alterations, and co-occurring amplifications (with the exception of the co-occurring amplification analysis) were excluded from the molecular landscape analysis. Amplifications for *FGFR2* were detected based on plasma copy number >2 (low = 2.1-2.4; medium = 2.4-4.0; high = >4.0). Co-occurring alterations detected via ctDNA were evaluated for patients who harbored *FGFR2*_amp_ and compared to those with *FGFR2*_Null_. Fisher’s exact test was used for group comparisons, except one multivariate analysis that required a 2-way ANOVA (specific statistical test is noted in the figure legend). Significance is noted with asterisks (*P ≤ 0.05, **P ≤ 0.01, ***P ≤ 0.001, and ****P ≤ 0.0001).

### cBioPortal MSK cohort

To provide comparison and context for our ctDNA analyses, we interrogated the publicly available cBioPortal tissue databases (MSK cohorts; 2020 and 2022)^13-15^ to determine tissue-derived molecular profiles. EAC, GC, and GEJ adenocarcinomas of pathological and clinical stage IV were included. Esophageal poorly differentiated carcinoma samples were excluded. Samples with *FGFR2* amplifications were queried and assessed for co-occurring alterations, excluding co-occurring amplifications. Comparisons between patients with *FGFR2*_amp_ versus *FGFR2*_Null_ focused on genes that were also included on the G360 panel used for the ctDNA analysis. *FGFR2* amplification on tissue NGS was detected using a cutoff of copy number > 2.^16^ Fisher’s exact test was used for group comparisons.

### Guardant INFORM database

The INFORM DB real-world database was used for real-world performance validation.^17,18^ INFORM DB contains genomic information from more than 225 000 patients tested using the G360 ctDNA platform linked with US administrative claims data. A single unique person-level identifier is used to link the genomic test data and claims data. INFORM DB includes claims for reimbursement of privately insured patients and does not have records of Medicare reimbursed claims. Patients with EAC, GC, or GEJ adenocarcinoma who received at least one G360 test between June 2014 and September 2022 were included in the study cohort. Records of their treatments per standard guidelines were extracted from medical procedures and paid pharmacy claims data.

Line of therapy regimen information was summarized by combining all new anticancer drugs that started within 21 days, and any new anticancer drugs started outside of this 21-day window indicate a new line of therapy. Dropping drugs from a regimen does not indicate a new line. Real-world overall survival (rwOS) is defined as the time between the initiation of first-line therapy to death. Patients without a date of death were censored at the date of their last known activity. Time to discontinuation (TTD) is defined as the time between the initiation of first-line therapy to discontinuation of first-line therapy or death while receiving first-line therapy. Time to next treatment (TTNT) is defined as the time between the initiation of first-line therapy to the initiation of second-line therapy or death while receiving first-line therapy. Patients without evidence of discontinuation or second-line treatment were censored at the date of their last known activity.

### Multi-institutional clinical data

In an effort to have patient-level data including demographics, treatment and survival in *FGFR2*_amp_ GECs, we identified 4 high volume cancer centers (Moffitt Cancer Center, Memorial Sloan Kettering, University of California Irvine and Massachusetts General Hospital) performing G360 tests in GECs. We identified 31 patients with *FGFR2* amplification detected via G360 and who were diagnosed between 2016 and 2022. We present clinical data using descriptive statistics (median estimates for continuous variables and percentages for categorical variables). OS was calculated for each patient from time of diagnosis to death due to any cause. Median estimates for survival were provided.

## Results

### Unique co-occurring alterations were enriched in FGFR2_amp_ cases on a liquid biopsy platform

**Figure 2.**
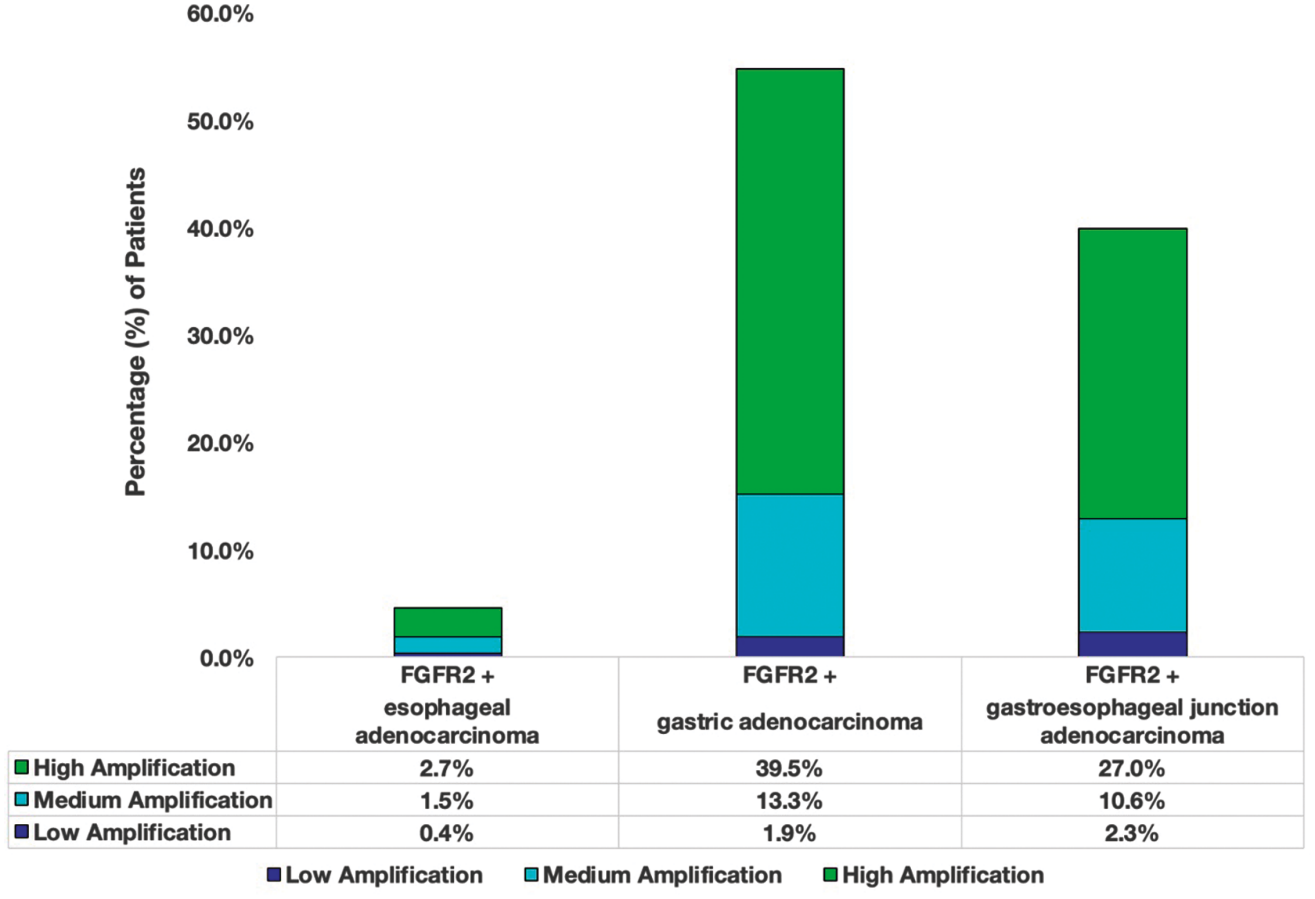
Distribution of *FGFR2*_amp_ GECs based on the location of the tumor as noted in the Guardant 360 ctDNA cohort. The different colors define the degree of amplification. Most amps were high (+++) in GC and GEJ (plasma CN ≥ 4).

Approximately 7100 patients from the Guardant Health database that met the diagnosis criteria were evaluated. From this cohort, 263 (3.7%) patients harbored *FGFR2*_amp._ Median age was 66 years, with majority being males (65% males versus 34% females). *FGFR2* status was stratified based on cancer type. Among patients with *FGFR2*_amp_ cancers, GC and GEJ cancers were observed to have a higher percentage of high (+++) *FGFR2* amplifications (39.5% [104/263] and 27% [71/263] for GC and GEJ cancers, respectively; Figure 2; Supplementary Figure S1). The *FGFR2* amplifications were more frequently observed in patients who were tested at diagnosis (44%, 116/263) versus those with prior treatment (19%, 49/263; *P* = .0147). Diagnosis data were unavailable for 37% of patients with *FGFR2*_amp_ (98/263).

**Figure 3.**
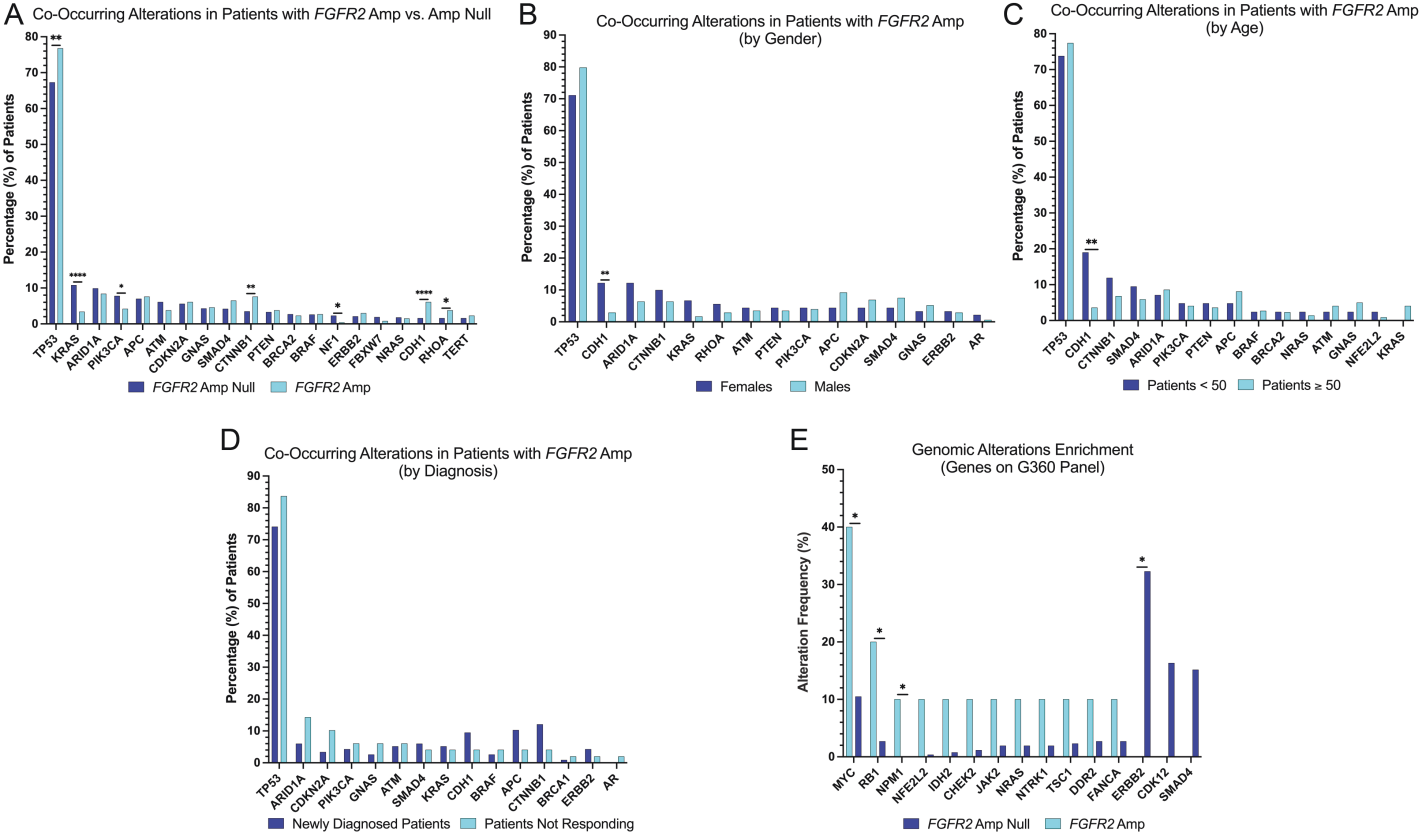
Co-occurring alterations in *FGFR2*_amp_ and *FGFR2*_null_ from Guardant Health Database. (A) Co-occurring alterations for *FGFR2*_amp_ versus *FGFR2*_null_ in the entire cohort. (B) Co-occurring alterations in *FGFR2*_amp_ GECs by gender. (C) Co-occurring alterations in *FGFR2*_amp_ by age. (D) Co-occurring alterations in *FGFR2*_amp_ by the timing of test. (E) Comparison of co-occurring alterations by *FGFR2* status in the MSK cohort.

Co-alterations were evaluated for patients with EAC, GC, and GEJ cancers who harbored *FGFR2*_amp_ (amp vs null) via ctDNA. Patients who harbored *FGFR2*_amp_, were found to be enriched for co-occurring SNVs (single nucleotide variants) in *TP53* (*P* = .0012), *CTNNB1* (*P* = .0018), *CDH1* (*P* < .0001), and *RHOA* (0.0112), while also harboring significantly less frequent mutations in *KRAS* (*P* < .0001), *PIK3CA* (0.0325), and *NF1* (*P* = .0316; Figure 3A). Among patients with *FGFR2*_amp_, *CDH1* (*P* < .0001) was significantly enriched among females (Figure 3B) and patients under the age of 50 years (*P* = .0011; Figure 3C). There were no statistically significant difference among co-occurring alterations based on cancer type (Supplementary Figure 2). There was also no statistically significant alterations among patients with *FGFR2*_amp_ cancers in those who were newly diagnosed versus those not responding to therapy (Figure 3D), suggesting a similar frequency of amplification before and after therapy. A separate analysis of co-occurring amplifications was also assessed and *EGFR* (*P* = .008), *CCNE1* (*P* = .0004), *ERBB2* (*P* = .0355), and *AR* (*P* = .0324) amplifications were significantly enriched in patients with *FGFR2*_amp_ compared to *FGFR2*_Null_ (Supplementary Figure S3).

### Co-occurring mutations in FGFR2_amp_ cases using tissue NGS may not overlap with findings from liquid biopsy platform

Using MSK cohort tissue database, we further assessed for co-occurring alterations amongst patients diagnosed with stage IV GECs with *FGFR2*_amp_ versus *FGFR2*_Null_. Two hundred sixty-seven patients met the inclusion criteria. Ten (4%) harbored *FGFR2*_amp_ and 257 patients were *FGFR2*_Null_. Mutations in *MYC* (*P* = .0191), *PRKCI* (*P* = .0209), *NSD3* (*P* = .0416), *RB1* (*P* = .0396), *NPM1* (*P* = .0375), *PEAR1* (*P* = .0375), and *TACC2* (*P* = .0375) were enriched in *FGFR2*_amp_; and *ERBB2* (*P* = .0223) being the most common amongst patients with *FGFR2*_Null_ (Figure 3E).

### Real-world data mimics findings regarding FGF2_amp_ patients as that seen in genomic analysis

The Guardant INFORM DB identified 7492 patients with GECs who had a G360 assay performed between 2017 and 2022. Two hundred sixty-six (3.6%) patients harbored *FGFR2*_amp_; and 7226 patients (96.4%) were *FGFR2*_Null_ (as noted in the G360 genomic analysis, 3.7% patients harbored *FGFR2*_amp_). In the RWD cohorts (*FGFR2*_amp_ vs *FGFR2*_Null_), approximately 27% of patients had treatment information (72 patients and 1989 patients in *FGFR2*_amp_ and *FGFR2*_null_ cohorts, respectively). Among 2061 patients with treatment data, the median age was 63 years, the majority of patients were male (72.2%) and 59% of them were former or current smokers and these were similar when stratified by *FGFR2* status. The mean Elixhauser Comorbidity Index (ECI)^19^ was 6.82 and 6.37 for patients in *FGFR2*_amp_ and *FGFR2*_Null_ cohorts, respectively.

More patients in the *FGFR2*_amp_ cohort were treated with a 5-fluorouracil-based regimen (72.2%) compared to patients in *FGFR2*_null_ cohort (59.3%; Supplementary Table S1). Among patients in *FGFR2*_amp_ cohort, 34.72% had a G360 test performed before initial treatment and 70.83% on/or after first-line treatment. When looking at the alteration values among patients in *FGFR2*_amp_ cohort, we found that 75.56% of G360 tests (68/90 tests) had high amplifications (copy number > 4; Supplementary Table S2). Co-occurring mutations were also assessed in *FGFR2*_amp_ versus *FGFR2*_Null_ cohorts who received treatment. *TP53* was the most common co-occurring alteration detected both in *FGFR2*_amp_ and *FGFR2*_Null_ cohorts (81.94% and 62.04%, respectively; Supplementary Table S3).

**Figure 4.**
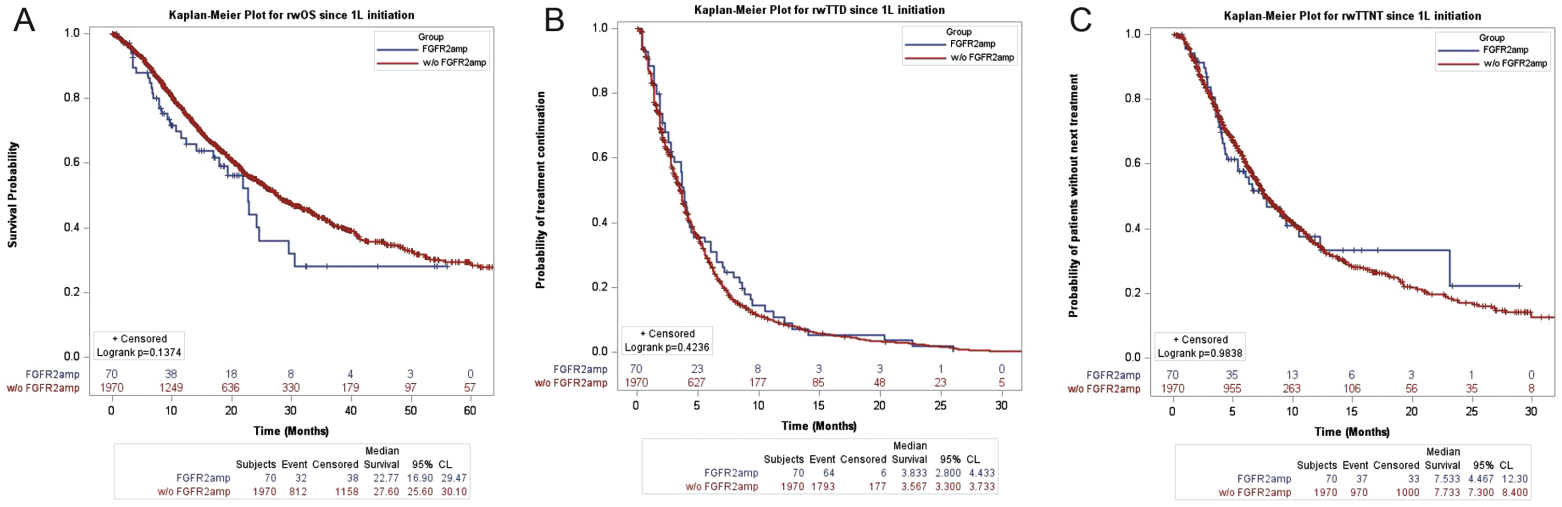
Clinical outcomes after first-line treatment in patients with *FGFR2*_amp_ versus *FGFR2*_null_, INFORM DB. (A) Comparison of TTD after first-line treatment in *FGFR2*_amp_ and *FGFR2*_null_ subgroups. (B) Comparison of rwOS since first-line treatment initiation by *FGFR2* status. (C) Comparison of rwTTNT after first-line treatment by *FGFR2* status.

No statistically significant differences were noted in rwOS, rwTTNT, and rwTTD between *FGFR2*_amp_ and *FGFR2*_Null_ cohorts (Figure 4). After interrogating the claims database, 5 patients had a claim for FGFR2-targeted treatment with erdafitinib (small-molecule inhibitor against FGFR1-4), of whom 2 patients had received the medication.

### Clinically FGFR2_amp_ cases represent a unique subset of GECs

A total of 31 patients with *FGFR2* amplification were evaluated. The majority of patients were diagnosed with Stage IVb adenocarcinoma (74.2%), with the lower third of the esophagus being the primary tumor site and approximately half involving the GEJ. The majority (14 of 31) of patients had multiple areas of metastatic disease (53.8%) and liver (19.2%) being the next most common single site of metastasis. Of the patients with metastatic disease, 84% received treatment. Of the 29 patients who had MMR testing performed, 28 (96.6%) were MMR proficient. One patient was tested as MMR deficient. HER2 test results were available for 27 patients (16% [5 of 31] were HER2 positive and 71% [22 of 31] were HER2 negative). HER2 positivity was defined as IHC (immunohistochemistry) 3+ or IHC 2+ and in situ hybridization amplified. PD-L1 test results were available for 23 patients, 48.4% (15 of 31) had a PD-L1 combined positive score (CPS) > 1. Most had metastases to multiple sites and received <3 lines of treatment. Among treatments received, most did not receive any form of FGFR-directed therapy, and only 7 patients (22.6%) received targeted therapy. Based on NGS, 7 of 17 (41%) patients had *FGFR2* amplification only without the presence of any other co-occurring mutations or fusions and 2 of 17 (11%) were detected to have both an amplification and fusion in *FGFR2*. Complete follow-up data were available for 20 patients of which 19 were deceased at the last follow-up and one was alive. The median survival for this cohort was 13.1 months (Table 1).

**Table 1.** Characteristics of selected patients with *FGFR2* amplification in the multi-institutional clinical cohort.

Characteristics	Result
Age (years), median (range)	62 (33-80)
Stage at diagnosis (*n* = 31)	
Stage III	4 (12.9%)
Stage IVa	4 (12.9%)
Stage IVb	23 (74.2%)
Primary site of the tumor (*n* = 31)	
Lower third of esophagus	14 (45%)
Cardia of the stomach	5 (16%)
Fundus of the stomach	2 (6.5%)
Body of the stomach	6 (19.4%)
Antrum of the stomach	2 (6.5%)
Unknown site	2 (6.5%)
Tumor involved gastroesophageal junction (*n* = 31)	
Yes	15 (48.4%)
No	16 (51.6%)
Histology (*n* = 31)	
Adenocarcinoma	30 (96.8%)
Mixed	1 (3.2%)
Differentiation (*n* = 31)	
Moderately differentiated	10 (32.3%)
Poorly differentiated	20 (64.5%)
Unknown	1 (3.2%)
Metastatic disease type (*n* = 28)	
Recurrent	5 (17.9%)
De novo metastatic	23 (82.1%)
Received treatment for metastatic disease (*n* = 31)	
Yes	26 (84%)
No	5 (16%)
MMR status (*n* = 31)	
Proficient	28 (90.3%)
Deficient	1 (3.2%)
Not tested	2 (6.5%)
HER2 status (*n* = 31)	
0 by IHC and/or not amplified by ISH	15 (48.4%)
0 by IHC, but amplified by ISH	1 (3.2%)
1+ by IHC and/or not amplified by ISH	4 (12.9%)
2+ by IHC and amplified by ISH	2 (6.5%)
2+ by IHC and not amplified by ISH	2 (6.5%)
3+ by IHC	3 (9.7%)
IHC not available, but non-amplified by ISH	1 (3.2%)
No testing available	3 (9.7%)
PD-L1 CPS score (*n* = 31)	
CPS < 1	8 (25.8%)
CPS 1-4	5 (16.1%)
CPS 5-9	3 (9.7%)
CPS ≥ 10	7 (22.6%)
Not available	8 (25.8%)
Site of metastatic disease (*n* = 26)	
Lymph nodes	2 (7.7%)
Liver	5 (19.2%)
Peritoneum	2 (7.7%)
Bones	2 (7.7%)
Lung	1 (3.8%)
Mixed	14 (53.8%)
Received more than 3 lines of treatment (*n* = 31)	
Yes	7 (22.6%)
No	18 (58.1%)
Unknown	6 (19.4%)
Received FGFR-directed treatment (*n* = 31)	
Yes	7 (22.6%)
No	20 (64.5%)
Unknown	4 (12.9%)
Tissue next-generation sequencing performed (*n* = 31)	
Yes	17 (54.8%)
No	9 (29.0%)
Unknown	5 (16.2%)
FGFR alteration detected on tissue NGS (*n* = 17)	
* FGFR2*_amp_ only	7 (41.2%)
* FGFR2*_amp_ and fusion	2 (11.8%)

## Discussion

Tumoral molecular heterogeneity at a given time or over time, limits the efficacy of emerging treatment options. Sometimes lack of tissue limits the use of tissue NGS for every diagnosis of advanced malignancy. ctDNA assays complement tissue NGS as ctDNA has demonstrated to report a higher incidence of amplifications in RTKs. In a sub-analysis of the SCRUM-Japan and GOZILA studies, ctDNA was observed to identify *FGFR2*_amp_ in patients with GC who did not have an *FGFR2*_amp_ identified via tissue^20^ (*n* = 6). Patients who only had *FGFR2*_amp_ identified by ctDNA, and were treated with FGFR inhibitor therapy, did receive clinical benefit. In the TiFFANY study, a phase II basket trial of all solid tumors with *FGFR* alterations, 4 patients with GC (all *FGFR* amplifications) and 2 patients with esophageal cancers (1 mutation and 1 fusion) were treated with the pan-FGFR inhibitor futibatinib. *FGFR* alterations were determined using ctDNA. Three of the 4 patients with GC had stable disease to partial response. The study concluded that futibatinib does demonstrate efficacy in *FGFR-*altered refractory solid tumors.^21^ On the contrary, in the FIGHT study, overall 96% of patients were tested to overexpress FGFR2b on IHC; 17% had FGFR2 amplification on ctDNA, and only 13% tested positive using both methods. There was PFS and OS benefit with bemarituzumab in patients who had FGFR2b IHC expression, but the numbers were too small to make meaningful interpretation for benefit in the patients eligible based on ctDNA amplification alone.^11^

In our study, we found a similar prevalence of *FGFR2*_amp_ status when we compared ctDNA results to existing data from tissue NGS using the MSK cohort as well as to our real-world cohort, with approximately 4% of GECs harboring an *FGFR2*_amp_. This is consistent with results reported in other studies looking at relevance of ctDNA versus tissue in evaluating for *FGFR2*_amp_ in GECs.^22,23^ In prior studies, amplifications in *FGFR2* are estimated to be present in 5%-7% of GECs.^4,5^ Compared to historical data, our patient cohort demonstrated *FGFR2*_amp_ in 3.7% of patients, which could be an underestimation related to sample size, disease burden, or poor ctDNA shedding in some cases. Patients enriched with *FGFR2*_amp_ were most frequent with GC and GEJ cancers (40% and 27%, respectively). When looking across the overlap of key biomarkers in this space, in our clinical cohort, we identified 16% of patients as HER2 positive by standard clinical criteria. Another study identified 26% (7/27) of HER2 positive (IHC 3+) GC cases to have any FGFR2 staining (1+ to 3+).^24^ Also, in our cohort, 48.4% of patients were PD-L1 positive with a CPS of 1 or more (32.3% were CPS ≥ 5) which is similar to 31% of patients identified to have FGFR2 positive, PD-L1 positive (CPS ≥ 5) in the NIVOFGFR2 study.^25^

The molecular classification of GC denotes *FGFR2* amplification in both chromosomal instability (CIN) and genomically stable (GS) subtypes. Based on the spectrum of co-occurring genomic alterations, our findings are consistent with the TCGA data.^26^ When evaluating for co-occurring alterations among patients with *FGFR2*_amp_, we see that these patients harbored SNVs in *TP53*, *CTNNB1*, *CDH1*, *and RHOA* (Figure 3A). *CDH1* and *RHOA* are more prevalent among GS tumors versus *TP53* which is more common in CIN tumors.^27^ The receptor tyrosine kinase (RTK)-RAS pathway alterations are more prevalent amongst CIN GECs. *FGFR2* amplification facilitates cell growth by upregulating other RTKs directly influencing other pathways like MAPK/PI3K/mTOR and PKC/GSK3β.^28^ One similarly affected pathway involves E-cadherin which is regulated via *CDH1*; gene mutations in *CDH1* lead to loss of function of E-cadherin which leads to alterations in cell-to-cell adhesion and cell structure. This pathway is also regulated via PI3K/AKT/MTOR. Similarities in downstream signaling seem to be affected by *TP53*, but not by *RHOA* or *CTNNB1* which are linked to Wnt/B-catenin pathways.^29,30^ The lack of signaling similarity between *FGFR2* and *RHOA* or *CTNNB1* allows for potential treatment failure and resistance to FGFR2 pathway inhibition. This allows for room to elucidate other targeted strategies that would help inhibit these extraneous pathways. Among esophageal histologic subtypes, approximately 3% of EACs are shown to have *FGFR2* alterations, compared to other histologic subtypes which do not seem to harbor *FGFR2* alterations.^30^ This is consistent with our data as seen in Figure 2, where we see approximately 3% of EAC harbor high *FGFR2*_amp_.

In the MSK cohort, 3.74% of patients harbored an *FGFR2*_amp_, yielding similar results to the G360 cohort (Figure 3E). There was no similarity in co-occurring alterations between G360 and MSK cohorts when stratified for *FGFR2* amplification status. In the MSK cohort, patients with *FGFR2*_amp_ were enriched for *MYC*, *RB1*, and *NMP1* alterations that were not noted on the G360 panel, which could be due to tumor heterogeneity or the low sample size of patients with *FGFR2*_amp_ in the MSK cohort from a single institution. Tumor heterogeneity can also be attributed to tissue versus ctDNA sampling. Patient-specific factors, germline mutations, differences in somatic mutation profile, and environmental factors manifests as intratumoral heterogeneity which can account for the differences we see between tissue and ctDNA genomics as described. The Real World Evidence/INFORM DB provided a look at *FGFR2*_amp_ by evaluating patients from claims data who had received a G360 ctDNA test “liquid biopsy” as a standard of care. Similar to our results from the G360 database, the INFORM DB noted 3.6% of patients harbor *FGFR2*_amp_. The survival estimates on first-line treatment from this database (Figure 4) demonstrate a median rwOS of 22.7 months for patients with *FGFR2*_amp_. These estimates are higher than that reported in the FIGHT study of 11 months for the control arm.^11^ The discrepancies noted in our findings could be related to potential incomplete claims data. The potential impact of immortal bias in the clinical genomics database also needs to be considered. Since there is no currently FDA-approved FGFR2 inhibitor for the treatment of *FGFR2*_amp_ GECs, its implication on real-world estimates is limited. Our clinical data demonstrated that patients with an *FGFR2*_amp_ had a median survival of approximately 13.1 months. As some of the trials targeting *FGFR2* amplification in GECs mature, it can be expected for this to change. Given the small sample size of our clinical cohort, an accurate estimation on the general population cannot be applied.

Circulating tumor DNA has provided a shift in cancer diagnostics allowing us to capture tumor heterogeneity and genomic evolution, providing prognostic value in esophageal cancer and GC.^31^ Clinically, ctDNA use is limited as it is not routinely performed by many clinicians, likely due to a lack of consensus on optimal DNA sampling time. The genetic panel used to sequence variant allele frequencies to evaluate for somatic mutations and monitor disease response is limited and does not often reflect the full genomic landscape of the cancer being studied.^31,32^ Although limitations exist, ctDNA remains a valuable asset in the management of GECs. Our analysis demonstrates the utility of ctDNA sequencing in advanced GECs for identifying tumor heterogeneity and treatment of advanced *FGFR2*_amp_ disease with similar results reported in previous studies looking at the utilization of ctDNA.^22^

## Conclusions

Compared to colorectal cancer, GECs express a higher genomic heterogeneity with each patient demonstrating unique molecular patterns; as a result, ctDNA profiling may provide a more accurate representation of a GEC genomic profile.^33^ The FIGHT study^11^ evaluated *FGFR2* overexpression by IHC and ctDNA. Currently, there are 2 frontline studies FORTITUDE 101 (NCT05052801) and FORTITUDE 102 (NCT05111626) that when completed are expected to provide important data regarding bemarituzumab and FGFR2-targeted therapy^34,35^ in GECs. When large-scale data from such studies assessing the correlation of ctDNA and tissue-based *FGFR2* detection becomes available, this will add to the knowledge we gathered in this study.

## Supplementary Material

oyae061_suppl_Supplementary_Figures_1-3

oyae061_suppl_Supplementary_Tables_1-3

## Data Availability

The data underlying this article cannot be shared publicly due to this being aggregate data of information generated through standard-of-care testing. The data will be shared on reasonable request to the corresponding author.

## Previous presentation

Parts of this study were presented at GI ASCO 2023 in San Francisco, CA (abstract #429).
