# Supplementary figures and images for: Circulating tumor DNA molecular analyses and real-world evidence outcomes of FGFR2 amplified gastroesophageal cancers
Source: Oncologist. 2024 Jun 21;29(8):672–80. doi: 10.1093/oncolo/oyae061 (PMC11299948; doi:10.1093/oncolo/oyae061)

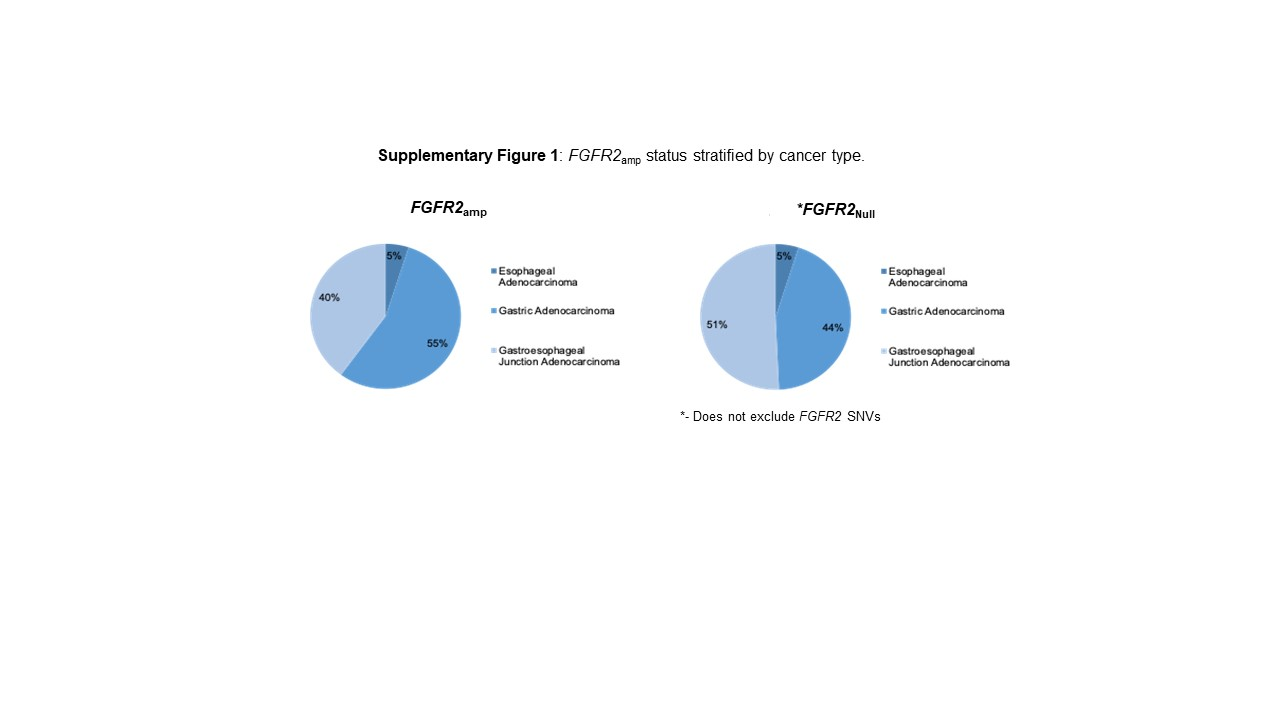


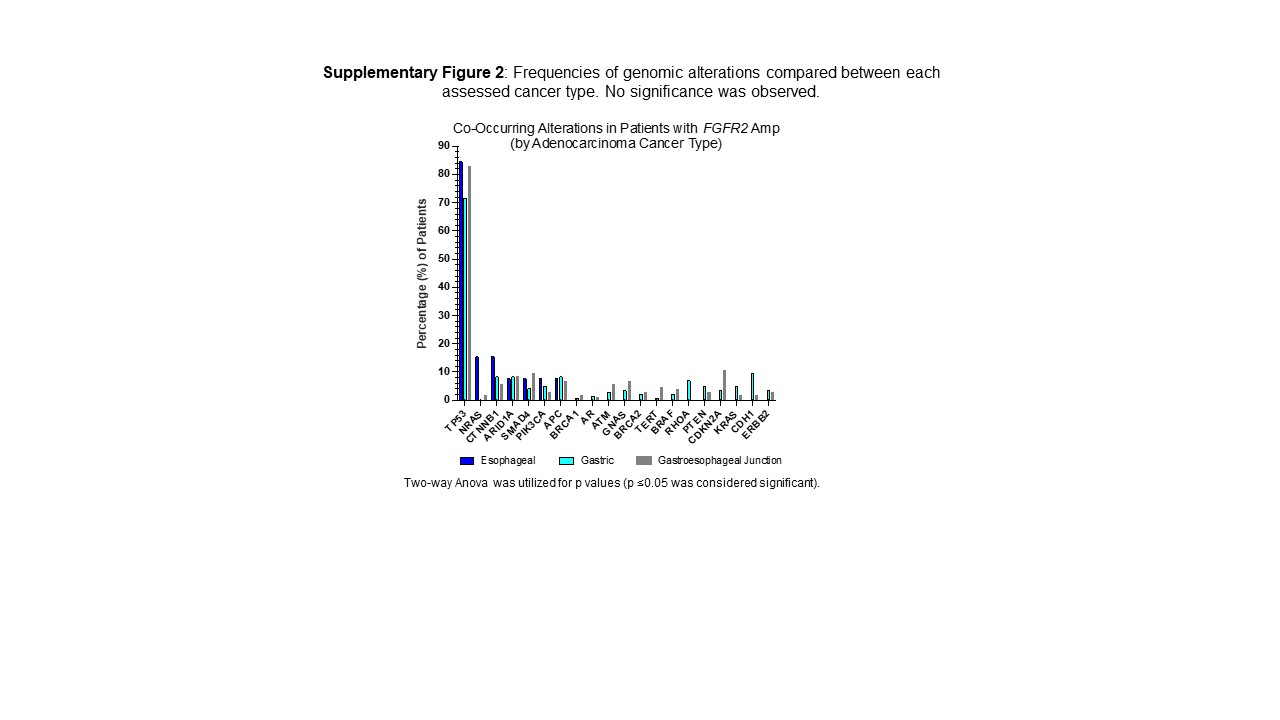


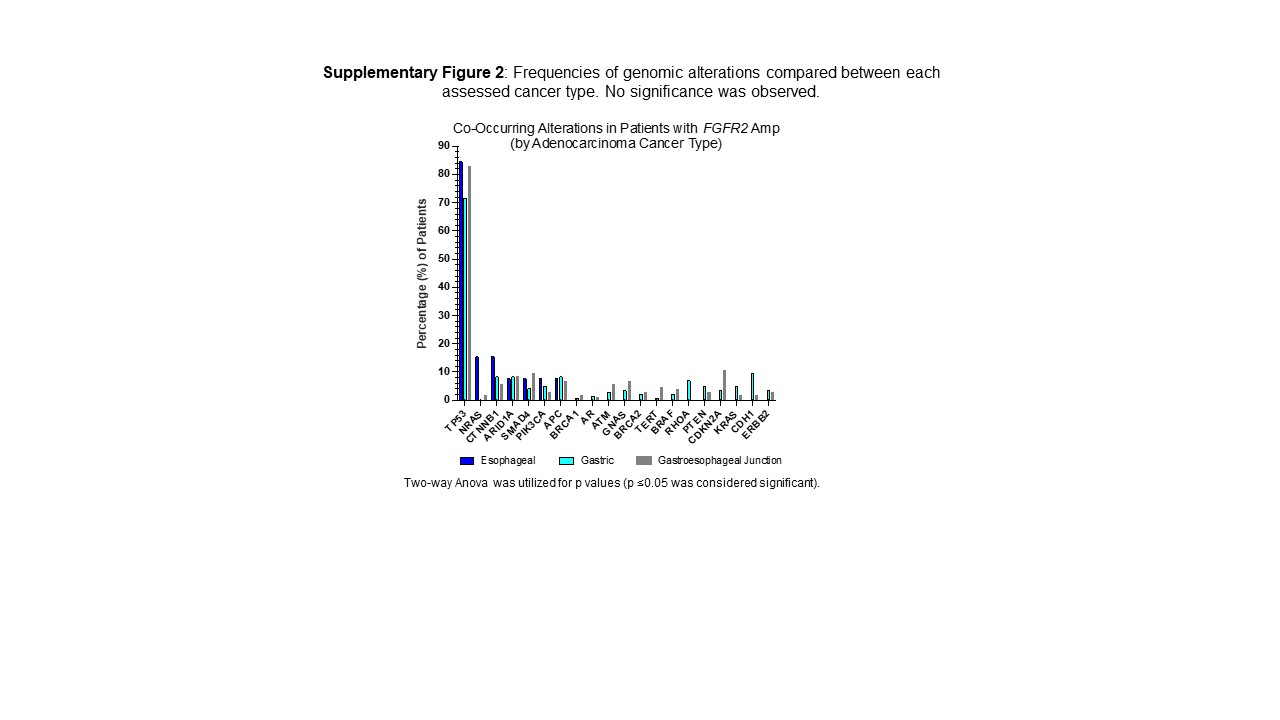

Supplement: oyae061_suppl_Supplementary_Figures_1-3 [file oyae061_suppl_supplementary_figures_1-3.docx]
