# Supplementary material for: Circulating tumor DNA molecular analyses and real-world evidence outcomes of FGFR2 amplified gastroesophageal cancers
Source: Oncologist. 2024 Jun 21;29(8):672–80. doi: 10.1093/oncolo/oyae061 (PMC11299948; doi:10.1093/oncolo/oyae061)
Supplement: oyae061_suppl_Supplementary_Tables_1-3 [file oyae061_suppl_supplementary_tables_1-3.docx]

**Supplementary Table 1:** First line treatment regimens for patients with *FGFR2*_amp_ *and FGFR2*_Null_

| **Patients with *FGFR2_a_*_mp_ (n=72)** | | | **Patients with *FGFR2*_Null_ (n=1,989)** | | |
| --- | --- | --- | --- | --- | --- |
| **Regimen** | **n** | **%** | **Regimen** | **n** | **%** |
| FOLFOX | 20 | 27.78 | FOLFOX | 596 | 29.96 |
| nivolumab + FOLFOX | 10 | 13.89 | carboplatin + paclitaxel | 152 | 7.64 |
| fluorouracil + oxaliplatin | 6 | 8.33 | trastuzumab + FOLFOX | 145 | 7.29 |
| trastuzumab + FOLFOX | 5 | 6.94 | docetaxel + FOLFOX | 113 | 5.68 |
| capecitabine | 4 | 5.56 | fluorouracil | 97 | 4.88 |
| docetaxel + FOLFOX | 4 | 5.56 | nivolumab + FOLFOX | 78 | 3.92 |
| fluorouracil | 3 | 4.17 | fluorouracil + oxaliplatin | 76 | 3.82 |
| carboplatin + paclitaxel | 2 | 2.78 | capecitabine | 74 | 3.72 |
| pembrolizumab | 2 | 2.78 | pembrolizumab | 68 | 3.42 |
| Unknown | 16 | 22.21 | unknown | 590 | 29.67 |

|  | **Patients with *FGFR2_a_*_mp_ (n=90 tests)** | | | | | |
| --- | --- | --- | --- | --- | --- | --- |
| Amplification status | **G360 test any time (n=90 tests)** | | **G360 test before 1L (n=27 tests)** | | **G360 test on/after 1L (n=63 tests)** | |
|  | n | % | n | % | n | % |
| High (+++) | 68 | 75.56 | 17 | 62.96 | 51 | 80.95 |
| Medium (++) | 20 | 22.22 | 9 | 33.33 | 11 | 17.46 |
| Low (+) | 2 | 2.22 | 1 | 3.7 | 1 | 1.59 |

**Supplementary Table 2:** Distribution of *FGFR2*_amp_ cases based on timing of treatment from INFORM DB cohort.

**Supplementary Table 3:** Co-occuring genetic alterations (amplifications included) comparing *FGFR2*_amp_ and *FGFR2*_Null_ from Guardant INFORM DB cohort.

| ***FGFR2_a_*_mp_ (n=72)** | | | ***FGFR2*_Null_ (n=1,989)** | | |
| --- | --- | --- | --- | --- | --- |
| **Gene** | **n** | **%** | **Gene** | **n** | **%** |
| *FGFR2* | 72 | 100.00% | *TP53* | 1234 | 62.04% |
| *TP53* | 59 | 81.94% | *KRAS** | 391 | 19.66% |
| *EGFR** | 25 | 34.72% | *EGFR** | 380 | 19.11% |
| *MYC* | 24 | 33.33% | *ERBB2** | 375 | 18.85% |
| *PIK3CA** | 24 | 33.33% | *PIK3CA** | 357 | 17.95% |
| *CDK6** | 19 | 26.39% | *ARID1A* | 288 | 14.48% |
| *MET* | 19 | 26.39% | *ATM* | 247 | 12.42% |
| *BRAF** | 16 | 22.22% | *MYC** | 244 | 12.27% |
| *ERBB2** | 15 | 20.83% | *MET** | 243 | 12.22% |
| *APC* | 14 | 19.44% | *APC* | 215 | 10.81% |
| *KRAS** | 13 | 18.06% | *CCNE1** | 215 | 10.81% |
| *ATM* | 12 | 16.67% | *CDK6** | 211 | 10.61% |
| *FGFR1* | 12 | 16.67% | *BRAF** | 179 | 9.00% |
| *CCNE1** | 11 | 15.28% | *SMAD4* | 148 | 7.44% |
| *CDKN2A* | 11 | 15.28% | *GNAS* | 143 | 7.19% |

Genes with amplification, denoted with *
